# What can we do with a dental avulsion? A multidisciplinary Clinical Protocol

**DOI:** 10.4317/jced.57198

**Published:** 2020-10-01

**Authors:** Naia Bustamante-Hernández, Jose Amengual-Lorenzo, Lucía Fernández-Estevan, Alvaro Zubizarreta-Macho, Cátia-Gisela Martinho da Costa, Rubén Agustín-Panadero

**Affiliations:** 1 Post Graduate Student in Buccofacial Prosthetics, Department of Stomatology, Faculty of Medicine and Dentistry, University of Valencia, Valencia, Spain; 2 Associate Professor, Department of Stomatology, Faculty of Medicine and Dentistry, University of Valencia, Valencia, Spain; 3 Associate Professor, Department of Implant Surgery, Faculty of Health Sciences, Alfonso X el Sabio University, Madrid, Spain; 4 Private practice, Valencia, Spain; 5 Adjunct Professor, Department of Stomatology, Faculty of Medicine and Dentistry, University of Valencia, Valencia, Spain

## Abstract

**Purpose:**

The aim of this case report was to explain a multidisciplinary and conservative approach carrying out the replantation of an avulsed closed apex central incisor stored in dry conditions for a 16-hour period from the moment of trauma.

**Case Report:**

This report describes a case of a 28 year-old male who suffered contusion of the upper lip, avulsion of right upper central incisor, enamel cracks after trauma of left upper central incisor and upper left lateral incisor crown fracture due to an accident. Avulsed tooth was dry stored and it was replanted 16h after the trauma. The root was disinfected and the necrotic periodontal tissue removed, the endodontic treatment was done before replantation and a flexible splint was applied to tooth 13 to tooth 23. Two months later a contralateral tooth presented crown discoloration occurred due to pulp necrosis an endodontic treatment as well as bleaching were carried out. An esthetic restoration for lateral incisor crown fracture was also done. In the one year review the patient remains asymptomatic, with no signs of root resorption or ankylosis of the damaged teeth.

**Conclusions:**

A conservative approach of tooth with delayed reimplantation can be a stable and functional with the appropriate treatment procedures. A clinical protocol for patients and professionals for the treatment of the avulsed tooth is proposed.

** Key words:**Endodontics, dental avulsion, avulsed tooth protocol.

## Introduction

Dental avulsion is described as a complete displacement of a tooth from its socket in the alveolar bone, and it is one of the most traumatic dental injuries which originates exposure of the cells of the periodontal ligament to the external environment as well as disruption of the blood supply to the pulp (1-6); resulting in a ischemic damage to the pulp tissue and periodontal ligament tissue (7,8). Avulsion of permanent teeth is seen in 0.5% to 3% (9,10) or 1-11% (11,12) of all dental injuries depending on the studies, being maxillary central incisors the most frequently affected tooth (13,14).

The factors that most influence the prognosis and outcome of replantation of the avulsed tooth are tooth development as well as extra-alveolar storage time and medium type (15). An appropriate storage medium as Hank’s balanced salt solution, saline, milk or saliva ensures the viability of the periodontal ligament cells present on the root surface (6).

If the avulsed tooth has closed apices, it is recommended an elective root canal treatment if it is replanted (9,10,16-19). The treatment of choice is usually as follows: in most cases endodontic treatment is performed (10,20) and antibiotic is normally prescribed. The patient is also instructed in diet, recommending soft food, and oral hygiene (10). Once the tooth has been repositioned in the socket, it is normally splinted and occlusion relieved to prevent root resorption (10,21).

Post-traumatic complications can occur deferred over time and can affect not only the tooth but also the supporting structures. Those ones could be: root resorption, pulp canal obliteration or pulp necrosis as well as soft tissue injuries or bone fractures, being pulp necrosis the most common post-traumatic complication (22,23).

This case report explains a multidisciplinary and conservative approach carrying out the replantation of an avulsed closed apex central incisor stored in dry conditions for a 16-hour period from the moment of trauma, avoiding more complex and less immediate treatments such as implants, and giving conservative solution to complications that can occur deferred over time.

## Case Report

**Figure 1:**
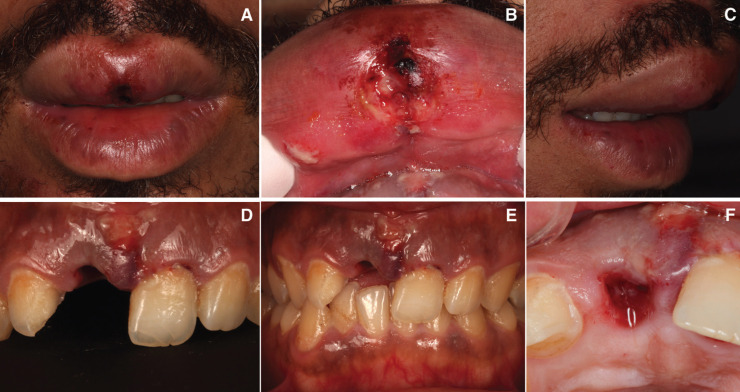
A,B,C) Upper lip contusion from different points of view. D,E) Avulsion of right upper central incisor. F) Socket healing after dental avulsion.

**Figure 2:**
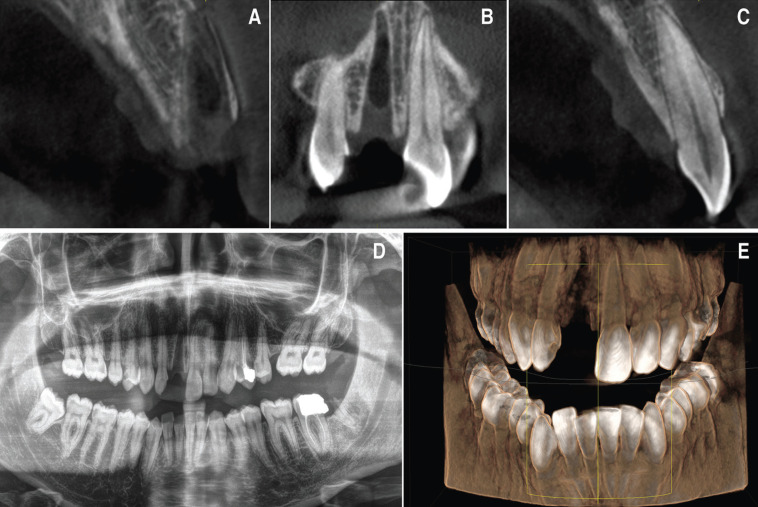
Pre-treatment radiographic study. A) Axial tomographic cut of the socket after the avulsion of the right upper incisor. B) Coronal view of the socket after the avulsion. C) Axial tomographic cut of the upper left incisor. D) Initial orthopantomography after tooth avulsion. E) 3D reconstruction of the patient’s bone jaws and teeth following dental trauma.

A healthy 28-year-old man visited the dental clinic with the upper right central incisor avulsed 16 hours after an accidental fall. The patient went to the hospital’s medical emergency services and there his soft tissues and mucous membranes were disinfected, but did not reposition the avulsed tooth. The tooth had been kept in milk, except for the two hours prior to the clinical visit. The patient delivered it on a paper napkin and completely dehydrated. After the clinical examination, photos and Cone Beam Computed Tomopraphy (CBCT) scan it was observed contusion of the upper lip (Fig. 1A-C), avulsion of the right upper central incisor (Fig. 1D-F), enamel cracks after trauma of left upper central incisor and upper left lateral incisor crown fracture, according to Andreasen’s classification which was also confirmed by the X- ray periapical study, CBCT scan (WhiteFox, Acteón Médico-Dental Ibérica S.A.U.-Satelec, Merignac, France) with the following exposure parameters: 105.0 kilovolt peak, 8.0 milliamperes, 7.20 s, and a field of view of 15 × 13 mm. (Fig. 2) and both thermal (Endo-Ice; Coltène/Whaledent, Lezennes, France) and electrical pulp tests (Parkell; Edgewood, NY, USA). As for the alveolar classification was a type 1, presenting all the bone plate preserved.

**Figure 3:**
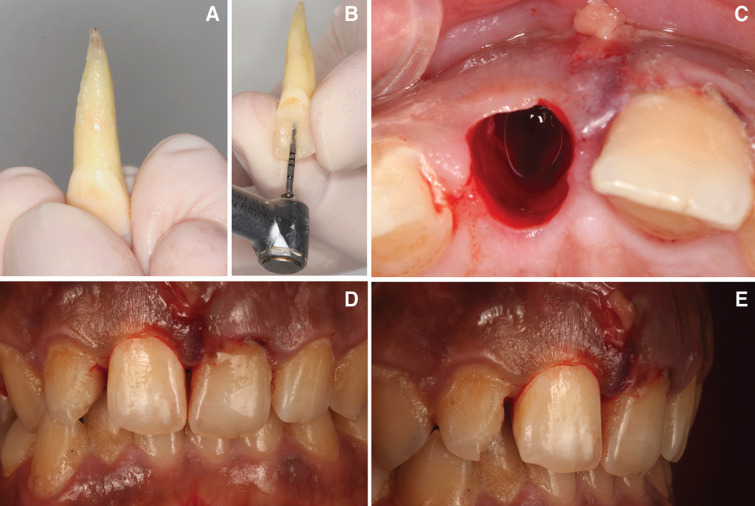
A) Avulsed tooth cleaned. B) Extraoral endodontic treatment. Access cavity preparation. C) Socket activated causing bleeding before intentional replantation. D,E) Tooth placed into his socket and splinted temporarily with orthodontic wire.

Initially, the avulsed tooth was cleaned carefully with sterile saline serum (Braun, Jaén, Spain) (Fig. 3A). During the clinical procedure the tooth was taken by its anatomical crown to prevent root surface damage. Subsequently, the endodontic access cavity was performed to allow a straight access to the root canal system. The working length of the root canal was established using a direct method, by subtracting 1mm from the actual root length determined by introducing a 10/.02 K-file (Dentsply Maillefer, Ballaigues, Switzerland) until it was visible through the apical foramen. Subsequently, extraoral root canal treatment was performed using a R25 reciprocating endodontic file (Reciproc; VDW, Munich, Germany) and irrigated with 5 ml of 5.25% sodium hypochlorite (NaOCl) (Clorox; Oakland, CA, USA), 5 ml of 17% ethylenediaminetetraacetic acid (EDTA) (SmearClear; SybronEndo, CA, USA), and 5 ml of sterile saline solution (Braun, Jaén, Spain) using an endodontic needle (Miraject Endo Luer; Hager & Werken, Duisburg, Germany) with a diameter of 0.3 mm inserted 1 mm into the working length (Fig. 3B). The contact between the irrigating solution and the surface of the root canal walls was enhanced by using an ultrasonic tip (IRRI S, VDW®, Munich, Germany). Afterwards, the root canal system was dried with sterile paper points (Dentsply Maillefer, Ballaigues, Switzerland) and finally, each root canal system was sealed using a warm gutta-percha system (Calamus, Dentsply Maillefer, Ballaigues, Switzerland) and an epoxy-amine resin-based sealer (AH Plus, Dentsply DeTrey, Konstanz, Germany) until the cemento-enamel junction. Furthermore, the access cavity was filled with a direct composite resin restoration (Filtek Supreme XTE, 3M™, MN, USA). Then, an apicectomy were carried out, removing the apical 3mm from the apex. A 3mm retrocavity was designed by means of an ultrasonic diamond tip (Ref.: PUSURG #2, ProUltra®, Dentsply Maillefer®, Ballaigues, Switzerland). and the retrocavity preparation was sealed with mineral trioxide aggregate as root-end filling material (ProRoot® MTA white, Dentsply Tulsa Dental®, Tulsa, UK).

**Figure 4:**
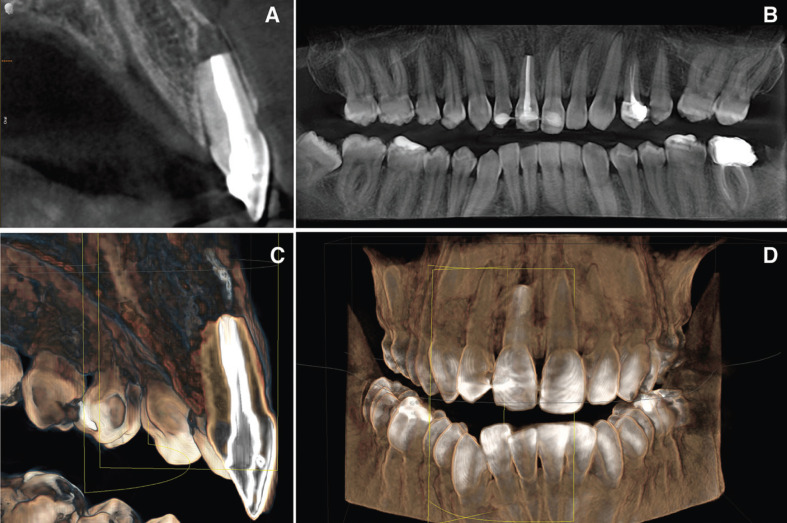
Radiographic study after replantation of the avulsed tooth. A) Axial tomographic cut of the upper right incisor replanted in the socket. B) Orthopantomography after tooth replantation. C) 3D axial reconstruction of the right upper incisor replanted. D) 3D reconstruction of the patient’s bone jaws and teeth after tooth replantation.

Replanted tooth was performed using infiltrative anaesthesia with lidocaine 2% and 1:100000 epinephrine (Artinibsa; Inibsa, Lliça de Vall, Barcelona, Spain). The alveolar socket was activated causing bleeding (Fig. 3C) before intentional replantation. The activation of the socket was carried out because the clot had already been established. The tooth was replaced into his alveolar socket (Fig. 3D,E) and splinted for 15 days with an orthodontic wire from 13 to 23 (Onlyorto, S.L., Barcelona, Spain) and composite resin (Filtek Supreme XTE, 3M™, MN, USA). Occlusal stops were also placed in tooth 4.6 and 3.6 to prevent occlusal contacts due to patient overbite. Antibiotic treatment was prescribed (Augmentine®, GSK,80 G, Brentford, United Kingdom) with analgesics (Dexketoprofen 25 mg, Menarini S.A., Barcelona, Spain), for one week. The patient was instructed in oral hygiene accompanied by chlorhexidine rinses and soft diet. The patient was scheduled for follow-up appointments at 1, 3, 6, 12 months in order to assess the clinical and radiographic evaluation of the treatment. At the 12 months follow-up visit, the patient remained asymptomatic and CBCT scan (WhiteFox, Acteón Médico-Dental Ibérica S.A.U.-Satelec, Merignac, France) was performed (Fig. 4).

**Figure 5:**
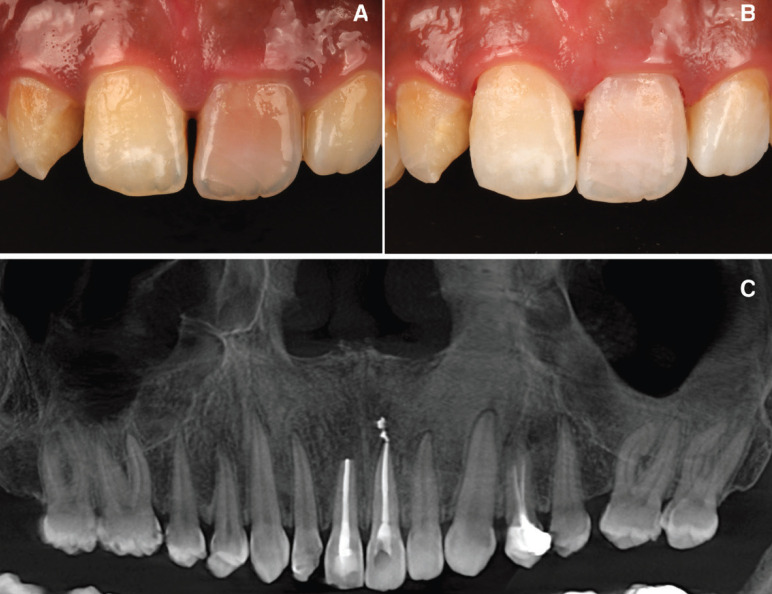
A) Contralateral tooth crown discoloration due to pulp necrosis. B) Contralateral crown bleaching due to crown discoloration after a pulp necrosis. C) Post treatment orthopantomography.

Three months after the replanted tooth, the upper left central incisor presented crown discoloration (Fig. 5A). The tooth was non-sensitive to both thermal (Endo-Ice; Coltène/Whaledent, Lezennes, France) and electrical pulp tests (Parkell; Edgewood, NY, USA). Periapical radiographs were taken to confirm the diagnosis of pulp necrosis. Root canal treatment was performed using infiltrative anaesthesia with lidocaine 2% and 1:100000 epinephrine (Artinibsa; Inibsa, Lliça de Vall, Barcelona, Spain). Rubber dam (Hygenic Dental Dam; Coltene Whaldent Gruppe, Altstätten, Switzerland) was disinfected with a povidone-iodine solution (Betadine; Meda, Solna, Sweden). Subsequently, the pulp chamber was opened to enable access to the root canal system. Root canal system instrumentation was performed with a 10/.02 K-file (Dentsply Maillefer, Ballaigues, Switzerland), and 1 ml of sterile saline solution (Braun, Jaén, Spain) was used to irrigate the canal and detach any bacteria adhered to dentin. The working length of the root canal system was determined using an electronic apex locator (Raypex 6; VDW, Munich, Germany) and verified with a working length radiograph using a 20/.02 K-file (Dentsply Maillefer, Ballaigues, Switzerland). The root canal system was prepared using an R25 reciprocating endodontic file (Reciproc; VDW, Munich, Germany) and irrigated with 5 ml of 5.25% NaOCl, 5 ml of 17% EDTA (SmearClear; SybronEndo, CA, USA), and 5 ml of sterile saline solution (Braun, Jaén, Spain) using an endodontic needle (Miraject Endo Luer; Hager & Werken, Duisburg, Germany) with a diameter of 0.3 mm inserted 1 mm into the working length. The contact between the irrigating solution and the surface of the root canal walls was enhanced by using an ultrasonic tip (IRRI S, VDW®, Munich, Germany). Afterwards, the root canal system was dried with sterile paper points (Dentsply Maillefer, Ballaigues, Switzerland) and finally, each root canal system was sealed using a warm gutta-percha system (Calamus, Dentsply Maillefer, Ballaigues, Switzerland) and an epoxy-amine resin-based sealer (AH Plus, Dentsply DeTrey, Konstanz, Germany) until the cemento-enamel junction.

**Figure 6:**
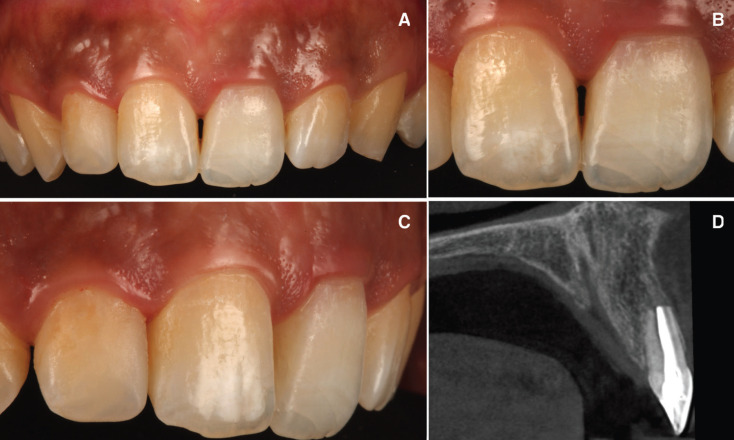
A,B,C) Post treatment clinical image. D) Axial tomographic cut of the upper right incisor in the bone socket after 1 year of treatment.

Furthermore, the access cavity was filled with a temporary restoration (Fermit, Liechtenstein, Ivoclar Vivadent). An internal-external bleaching of that tooth was subsequently carried out using 35% hydrogen peroxide (Philips Zoom WhiteSpeed, Royal Philips, Amsterdam, Holanda) combining with photoactivation using a LED lamp (Lamp Philips Zoom WhiteSpeed, Royal Philips), lasting 15 minutes in three applications. At the end of the first clinical session sodium perborate (Endoperox, Septodont) was placed inside the pulp chamber and it was sealed with the same provisional seal. Between clinical sessions had to pass 14 days and a second clinical session was held again, also of three photoactivated applications for fifteen minutes. Again sodium perborate was placed inside the pulp chamber for seven days and the temporary seal was put back (Fig. 5B,C). A week later it was checked if an harmonic tooth color had been reached with adjacent teeth and since this had been achieved, sodium perborate was removed again. In addition, a piece of cotton was placed inside the pulp chamber and a new temporary seal was made. Furthermore, the access cavity was filled with a direct composite resin restoration (Filtek Supreme XTE, 3M™, MN, USA). The patient was scheduled for follow-up appointments at 1, 3, 6 and12 months in order to assess the evaluation of the treatment. At the 12 months follow-up visit, the patient remained asymptomatic and periapical radiograph and CBCT scan (WhiteFox, Acteón Médico-Dental Ibérica S.A.U.-Satelec, Merignac, France) was performed. Six months after the intentional replantation the occlusal stops were removed (Fig. 6).

## Discussion

Dental avulsion treatment is complex (6), due to the fact that it is influenced by many different factors. The most influential factor is the periodontal ligament cells condition at the moment of the replantation, which in many cases are nonviable and are not expected to heal.

Unfortunately when such a traumatic accident occurs, an avulsed tooth manage is usually unknown (15). In this case patient was treated on the hospital’s medical emergency services but he did not receive any dental treatment, so the tooth was replanted 16h after trauma.

Due to the amount of hours passed and the inadequate treatment of the root surface and excessive dehydratation, the attached nonviable soft tissue was removed carefully (6,25). Thus, given the long delay in the replantation and the unfavorable storage conditions, this was a bad prognosis case. This is due to changes that occur in both the pulp and periodontal ligament, which will be decisive in the preservation or loss of the avulsed tooth. There are different clinical factors on which the treatment to be performed will depend: 1) time of tooth out of the mouth (extraoral period), 2) state of the periodontal ligament (conservation medium) and 3) degree of root development.

In this case, revascularization was not expected due to the closed apex. Extraoral root canal treatment is recommended in the International Association of Dental Traumatology (IADT) guidelines prior to the replantation of the tooth (6,25,26), and this is exactly what was done. Then, the apicoectomy was carried out and the root end was obturated with mineral trioxide aggregate (Proroot MTA, Dentsply Sirona, Bensheim, Deutschland), the socket was activated and the incisor replanted.

In these kinds of pathologies root resorption is considered a risk (10,16,17,27). Added to this is the increased risk from the bleaching treatment performed (28). Further risks include abscess formation, pulp necrosis and ankylosis. Complications can be deferred over time and other teeth that have been able to receive trauma may show pathological signs long after the accident, so they should be examined. Two months after the initial treatment contralateral tooth presented crown discoloration due to pulp necrosis. A conservative treatment was carried out, endodontic treatment as well as internal and external whitening were performed. In this way more invasive treatments such as veneers or crowns were avoided, respecting the dental structure to the fullest.

Although in this case they have not been observed, ankylosis of the root to the alveolar bone as well as root resorption are the most common complications after replantation (6,28,29). It should be considered that in adults, ankylosed teeth are able to remain functional for many years due, in particular, to the slow rate of bone remodeling (28,30-33). This is why it was decided to take the risk of ankylosis before performing a more invasive treatment such as implant replacement of the tooth.

Therefore, on the basis of the present clinical case, a conservative treatment protocol for the avulsed tooth is presented, depending on the extraoral time elapsed and the amount of root development:

If the extraoral time is short, less than one hour there can be cellular vitality, so treatment will depend on root development. The tooth must be manipulated by the crown to avoid further injuring the periodontal ligament, as well as it must be continuously moist. Only if the root surface appears contaminated should it be cleaned with physiological serum.

•When the apex is open, revascularization and apical closure are possible. Therefore, immature teeth with extraoral period less than one hour should be inserted into a doxycycline solution before being immediately replanted, which could improve revascularization.

•Although in teeth with closed apex there is no possibility of revascularization, if extraoral time is short, the chances of periodontal healing are accepTable. The steps to be performed are as follows: Cleaning the root surface with physiological serum, replant with slight pressure, because if it is strong it could affect the cells of the periodontal ligament and increase the probabilities of ankylosis.

When the tooth spends an extraoral time longer than sixty minutes, cell necrosis occurs. That is why in all cases endodontic treatment should be performed.

•If the tooth has an open apex when avulsion occurs a gentle cleansing of the periodontal ligament should be carried out. Subsequently performing an apexification treatment and a reimplantation.

•Instead if the avulsed tooth has the apex closed, after a gentle cleansing of the periodontal ligament it should be done an extraoral endodontic treatment and apicectomy using MTA and the consequent replantation (34).

In all cases if there is bone fracture, it should be attempted to reduce. As well as if there are lesions in the soft tissues, they should be sutured, especially in the cervical margin. In the same way in all cases oral hygiene instructions, chlorhexidine rinses, soft diet recommendations should be given. Antibiotic and analgesic treatment must also be provided. And ferulization of the avulsed tooth should be performed to prevent ankylosis (Table 1).

**Table 1:**
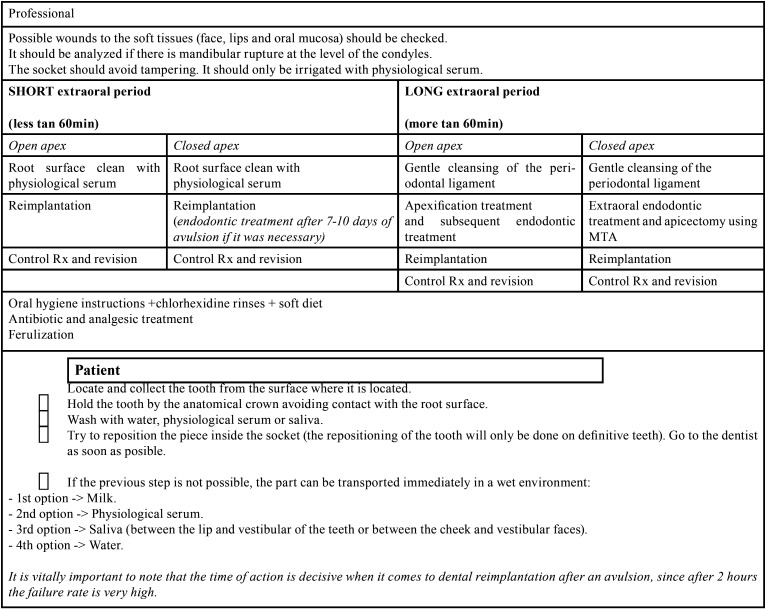
Avulsed tooth. Protocol. Guidelines for dentist and patient.

## Conclusions

In conclusion, despite a prolonged extra-alveolar dry storage time a conservative approach of tooth with delayed reimplantation can be a sTable and functional with the appropriate treatment procedures. It is proposed a clinical protocol for patients and professionals for the treatment of the avulsed tooth.
